# Egg retention, viviparity and ovoviviparity in Paraneoptera

**DOI:** 10.3897/CompCytogen.v15i3.70216

**Published:** 2021-07-31

**Authors:** Ilya A. Gavrilov-Zimin

**Affiliations:** 1 Zoological Institute, Russian Academy of Sciences, Universitetskaya nab. 1, St. Petersburg, 199034, Russia Zoological Institute, Russian Academy of Sciences St. Petersburg Russia

**Keywords:** Embryogenesis, oviposition, neoteny, paedogenesis

## Abstract

This article is a second part of the themed issue “Aberrant cytogenetic and reproductive patterns in the evolution of Paraneoptera insects”, prepared by the Russian-Bulgarian research team. Here, analysis of aberrations related to the egg development is provided based on literature data and the author’s own investigations. Evolutionary aspects of ovoviviparity/viviparity are also briefly discussed. Material and methods, terminology and nomenclature of taxonomic names are listed in the first paper of the issue (Gavrilov-Zimin et al. 2021).

In most Paraneoptera insects, embryogenesis starts only after the egg is laid outside the mother’s body. Cleavage is usually meroblastic being evidenced by the presence of a large amount of yolk in the egg. The zygotic nucleus undergoes divisions and gives origin to blastomeres and vitellophages (Fig. 1). Blastomeres migrate to the egg surface and form blastoderm. The vitellophages are few in number and dispersed between yolk drops. The blastoderm differentiates into serosa and germ band. The invaginations of the germ band into the yolk (anatrepsis) together with the intensive divisions of its cells lead to the emergence of the embryo and amnion. At maximal invagination, the germ band has a characteristic S-shaped form. When the invagination is finished, the inner germ band cells give rise to mesoderm and preliminary organogenesis starts. At the same time, buds of appendages appear and the embryo starts to turn backwards in comparison to its initial position (katatrepsis). The serosa degenerates whereas the amnion gives rise to the yolk epithelium. Finally, all yolk is consumed and the embryo achieves the size and form of the primolarva (Fig. 1). This general scheme of the embryonal development may have various modifications in different groups (see for reviews: Hagan 1951; Buchner 1965; Zakhvatkin 1975; Haga 1985).

**Figure 1.**
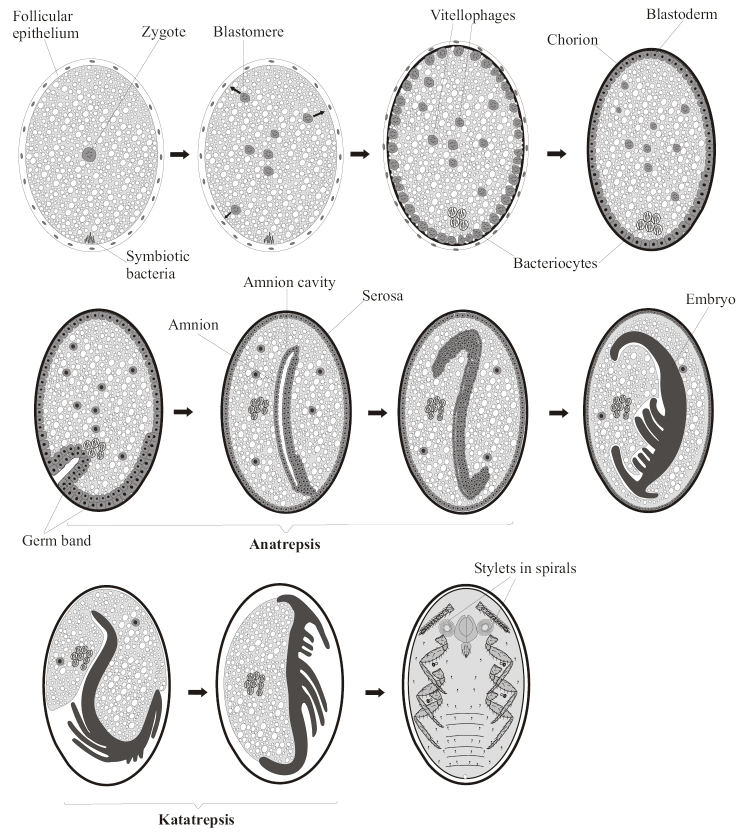
Main stages of typical Paraneoptera meroblastic embryogenesis (on the example of scale insects).

The modes of egg retention are diverse and originated many times in different phylogenetic lineages of Paraneoptera (Fig. 9). So, different variants of ovoviviparity and/or placental viviparity are observed even in the most archaic Paraneoptera, in some species of Copeognatha from the families Archipsocidae, Trogiidae, and Pseudocaeciliidae (Fernando 1934; Jentsch 1936; Mockford 1957; Wong and Thornton 1968). In the most studied viviparous psocid species, *Archipsocopsisfernandi* (Pearman, 1934), the whole embryonal development occurs inside the mother’s ovarium; the egg lacks a chorion and “yolk-cells”, but demonstrates a meroblastic cleavage (Fig. 2b); the serosa fuses with the wall of the ovarian tubule and forms a placenta-like organ for temporary nutrition of the embryo (Fig. 2b). It appears that all 18 species of the genus *Archipsocopsis* Badonell, 1948 are viviparous and lack gonapophyses, in contrast to the closely related and normally oviparous genus *Archipsocus* Hagen, 1882, whose females have gonapophyses (New 1987: 7). At least, some Parasita, for example, Mallophaga lice of the genus *Meinertzhageniella* Eichler, 1940, as well as Siphunculata lice *Polyplaxserrata* (Burmeister, 1839) and *Hoplopleura* sp. show ovoviviparous reproduction (Eichler 1946; Golub and Nokkala 2004), but the general picture of the ovoviviparity/viviparity in Copeognatha and Parasita is presently unclear because of the poorly studied reproductive patterns in most species of these groups.

**Figure 2.**
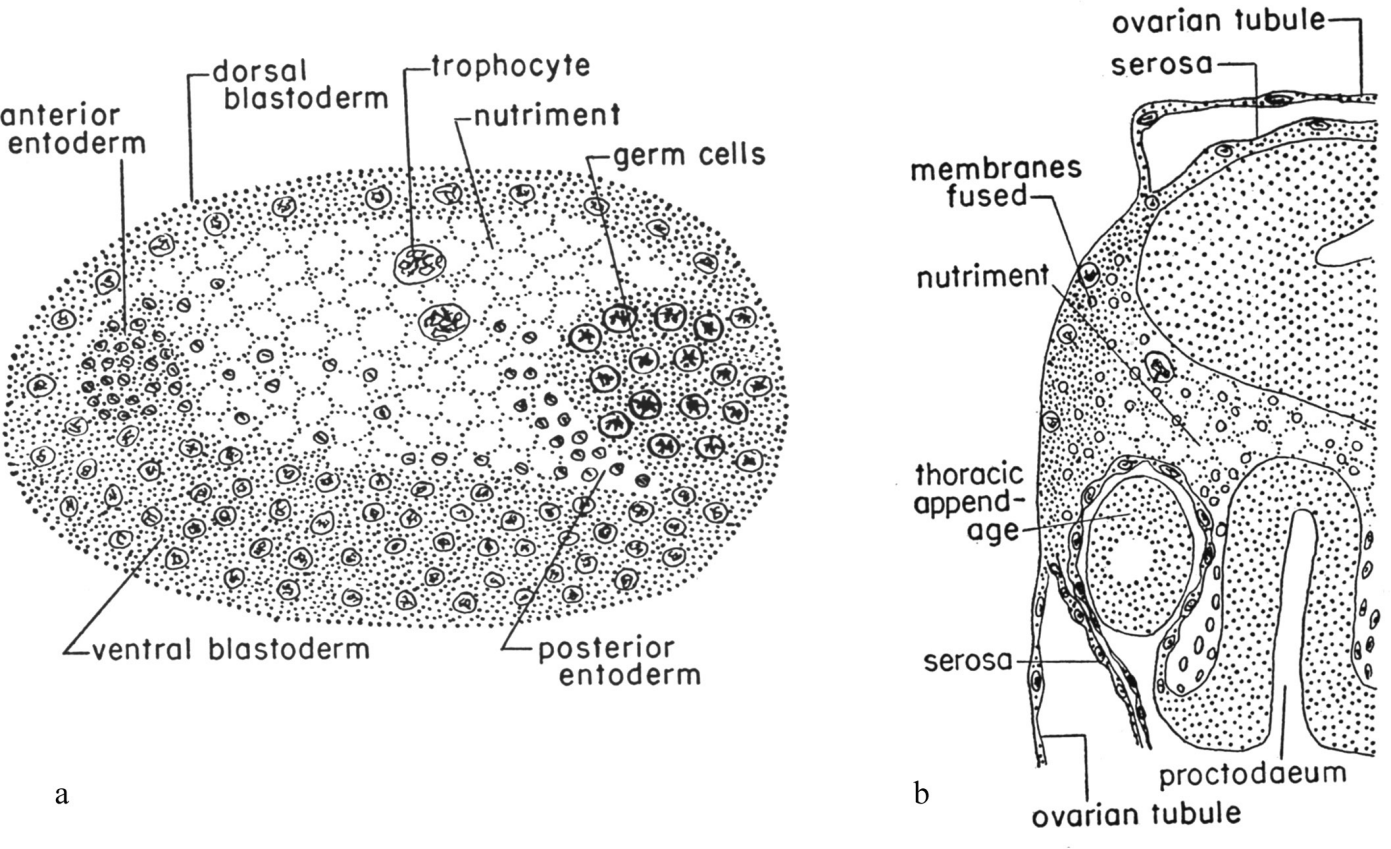
Embryonal development of *Archipsocopsisfernandi* (Copeognatha) (after Fernando 1934) **a** formation of endoderm (sagittal section) **b** fusion of serosa with the ovarian tubule to form a nutrient placenta-like organ (sagittal section).

In Thysanoptera, different (ovo)viviparous species were reported in the suborder Tubulifera, family Phlaeothripidae (e.g. Bagnell 1921; John 1923; Hood 1934: 71; Hathaway 1938; Bournier 1966), but in most of these reports, the authors did not provide a clear difference between viviparity and ovoviviparity. Some species of Tubulifera convincingly show facultative and incomplete ovoviviparity by laying eggs at different stages of embryogenesis (Viswanathan and Ananthakrishnan 1973; Ananthakrisnan and Dhileepan 1984; Dhileepan and Ananthakrisnan 1987; Nagrale 2012), which is similar to the same modes of oviposition in scale insects (see below).

It appears that no viviparous or ovoviviparous species have been found up to now in three suborders of Homoptera: Cicadinea, Psyllinea, and Aleyrodinea (Fig. 3), which combine together more than 50,000 recent species. Meanwhile, the phylogenetic lineage Aphidococca (suborders Aphidinea+Coccinea) shows numerous species, genera and families, which exhibit the embryonic development inside the mother’s body (Fig. 9).

**Figure 3.**
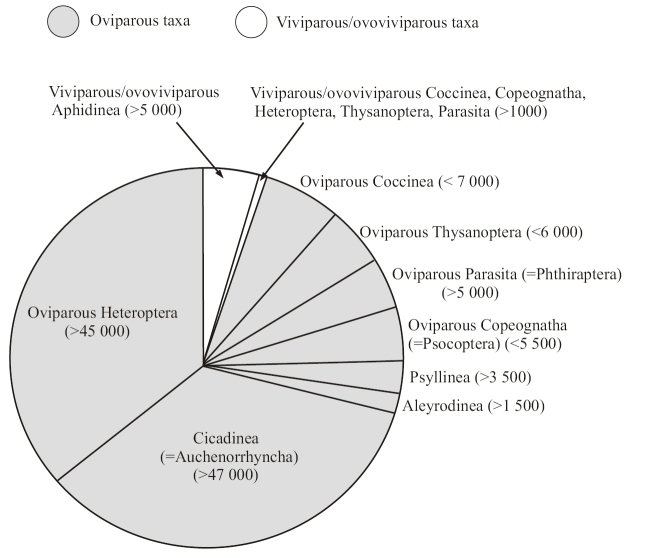
Approximate numbers of oviparous and ovoviviparous/viviparous species in different groups of Paraneoptera.

Among Aphidinea, only Adelgidae and Phylloxeridae (comprising together about 140 species in the world fauna) are obligately oviparous and even keep the ovipositor in adult females, whereas all other families (with about 5000 species) demonstrate obligate viviparity (rarely ovoviviparity) in parthenogenetic generations retaining oviparity in the bisexual generation only (Hille Ris Lambers 1950; Blackman 1987; Favret et al. 2016). It is interesting to note that all examples of aphid ovoviviparity were found by Hille Ris Lambers (1950) in the family Eriosomatidae (=Pemphigidae), i.e. in the most “primitive” group of “true aphids”. The uniqueness of the aphid viviparity lies in the very precocious start of the embryogenesis in the parthenogenetic egg, before the birth of the mother itself. Such eggs are very small, lacking yolk and chorion; the entire embryogenesis occurs inside the vitellarium, and the egg receives nutrition directly from the cells of the follicular epithelium (Fig. 4) (Uichanco 1924; Hagan 1951; Blackman 1987).

**Figure 4.**
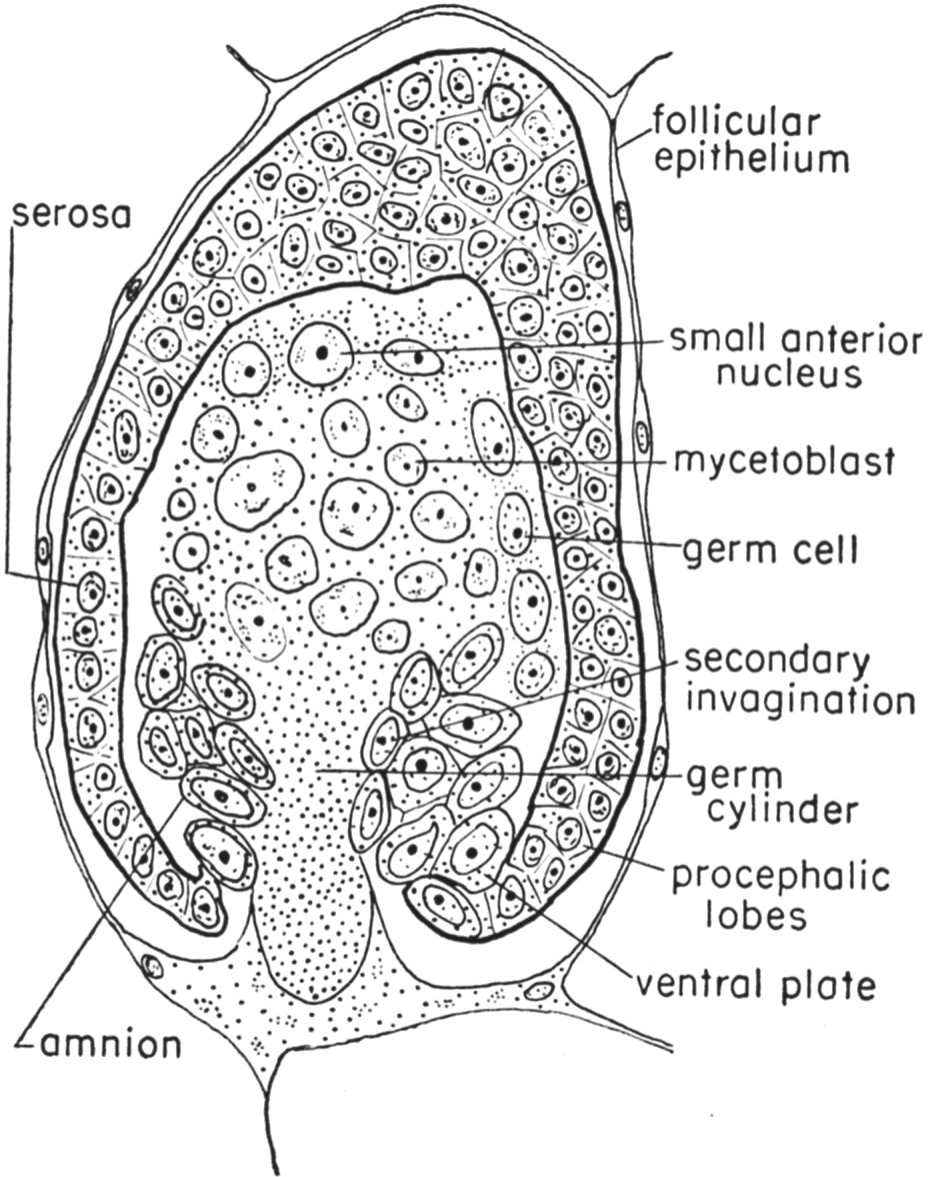
Developing embryo in vitellarium of a viviparous parthenogenetic aphid female of *Uroleucontanaceti* (Linnaeus, 1758) (after Uichanco 1924, with modifications of Hagan 1951).

The most diverse examples of egg retention and (ovo)viviparity are known in scale insects (see for review: Gavrilov-Zimin 2018). The most ancient and “primitive” scale insects from the tribes Matsucoccini and Steingeliini (family Margarodidae), are characterized by facultative ovoviviparity when the embryogenesis starts inside the mother’s body and, at least, some eggs undergo complete embryogenesis before oviposition, whereas other eggs of the same female are laid at early stages of the development. In such cases, the external incubation period (after the moment of oviposition) varies significantly from several days to one month (e.g., Bodenheimer and Harpaz 1955; McKenzie 1943). Obviously, this reproductive mode has originated in scale insect females as a result of neoteny and loss of the imaginal structures of the reproductive system (for example, ovipositor); i.e. facultative ovoviviparity can be considered as an apomorphy of Coccinea (Fig. 5) (Gavrilov-Zimin 2018). Probably, facultative incomplete ovoviviparity is also present in other archaeococcids of the tribe Xylococcini and, at least, in some Cryptokermesini (Margarodidae) (Vayssière and Hughes-Schrader 1948; Gavrilov-Zimin 2018). The small, morphologically aberrant archaeococcid families Xenococcidae, Phenacoleachiidae, and Carayonemidae are probably characterized by obligate ovoviviparity (Silvestri 1924, 1926; Gullan and Cook 2001; Kozár and Foldi 2001). On the other hand, at least some species of Kuwaniini, Coelostomidiini, and Margarodinae s.s. show normal oviparity with the beginning of the cleavage division after oviposition. The most diverse archaeococcid group, the Monophlebinae, is unfortunately very poorly studied in terms of embryology and reproductive biology, except only the tribe Iceryini (Hughes-Schrader 1948; Gavrilov-Zimin 2018). On the one hand, six Monophlebinae genera are characterized by the presence of a marsupium (Fig. 6) and, at least, in *Steatococcussamaraius* Morrison, 1927 eggs are laid in the marsupium just prior to katatrepsis suggesting that incomplete ovoviviparity occurs. Moreover, females of *Steatococcushystrix* Gavrilov-Zimin et Stekolshikov, 2018 (from Mali, Africa) contain the embryos with visible appendages even before the marsupial pouch is formed. On the other hand, at least some species of *Crypticerya* Cockerell, 1895 and *Icerya* Signoret, 1876 exhibit obligate complete ovoviviparity and lay fully developed embryos beneath the body (Gavrilov-Zimin 2018). Eggs of Ortheziidae (at least in such common species as *Ortheziaurticae* Linnaeus, 1758, *Newsteadiafloccosa* (De Geer, 1778), and *Insignortheziainsignis* (Browne, 1887)), are full of different inclusions and it is rather difficult to understand at which moment the cleavage starts, although it most likely happens after the oviposition (Gavrilov-Zimin 2018).

**Figure 5.**
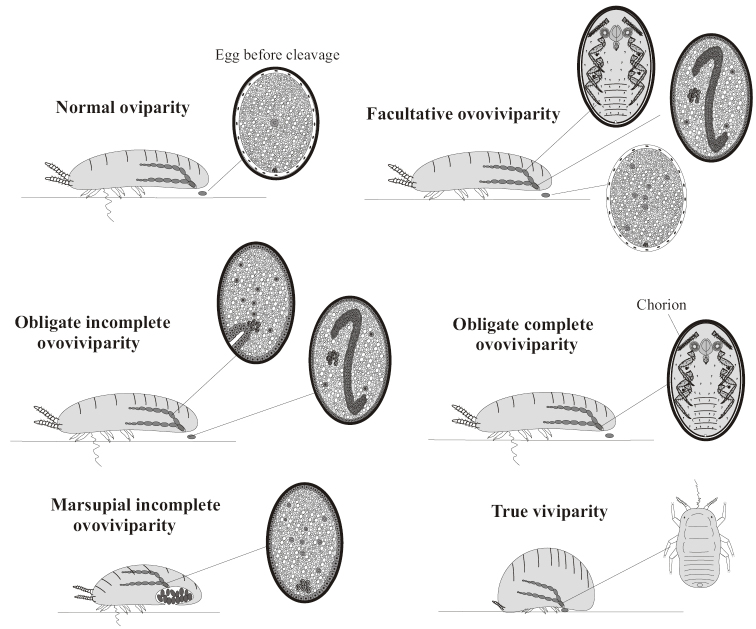
Different modes of reproduction in scale insects (Coccinea).

**Figure 6.**
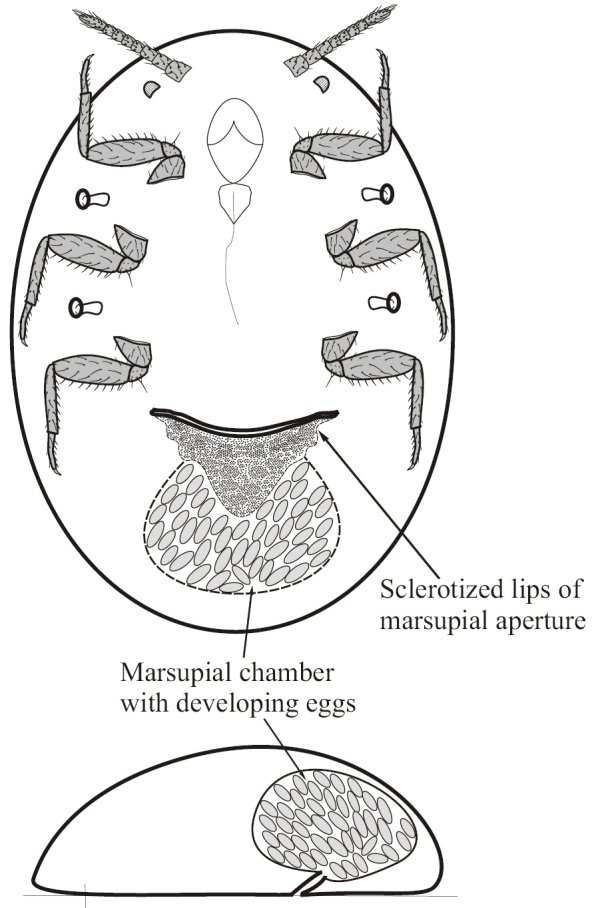
Schematic illustration of marsupium in *Etropera* spp. (Coccinea: Margarodidae) in vertical and horizontal projections.

The origin of the neococcid (superfamily Coccoidea) phylogenetic line was probably correlated with obligate complete ovoviviviparity (Gavrilov-Zimin and Danzig 2012; Danzig and Gavrilov-Zimin 2014; Gavrilov-Zimin 2018). This character was probably inherited by mealybugs (Pseudococcidae), the most primitive group of neococcids, from the obligate ovoviviparous ancestral family Phenacoleachiidae. It is known in numerous archaic genera of mealybugs including *Puto* Signoret, 1876, *Rastrococcus* Ferris, 1954, *Heliococcus* Šulc, 1912, *Fonscolombia* Lichtenstein, 1877, *Phenacoccus* Cockerell, 1893, *Paraputo* Laing, 1929, *Formicococcus* Takahashi, 1928 and many more divergent genera, for example all legless mealybugs (generic group *Antonina* Signoret, 1872), the species-rich genus *Mirococcopsis* Borchsenius, 1948, and numerous other small and monotypic genera. To date, more than 500 obligate ovoviviparous species of mealybugs from more than 60 genera have been reported, which is about 25% of the mealybug diversity in the global fauna. Moreover, there is no doubt that the real number of ovoviviparous mealybugs will increase due to further studies. A lot of species with complete ovoviviparity are known in other neococcid families: Eriococcidae, Micrococcidae, Coccidae, Aclerdidae, Dactylopiidae, Keriidae, Stictococcidae, Asterolecaniidae s.l., Beesoniidae, and Diaspididae (see: Gavrilov-Zimin 2018 for more detailed review). Viviparity in scale insects has been discovered till now only in three neococcid genera, including *Apiomorpha* Rübsaamen, 1894 (family Eriococcidae), *Stictococcus* Cockerell, 1903, and *Parastictococcus* Richard, 1971 (both from the family Stictococcidae). Eggs of the studied species from these genera are very small and yolk-poor; the developing embryo receives nutrition from the mother’s body through placenta-like structures (Buchner 1957, 1963, 1965) and does not have a chorion, which is always present in ovoviviparous species.

In general, the evolution of scale insects seems to show multiple cyclic conversions from oviparous reproduction to ovoviviparous/viviparous reproduction with the emergence of new peculiar adaptations for eggs’ protection (Fig. 7). Thus, in archaeococcids, the initial facultative ovoviviparity with the formation of loose ovisac (“primitive” genera of Xylococcinae s.l., most of Callipappinnae s.l.) evolves into normal oviparity in their probable descendants (Margarodinae s.s., some Monophlebinae and Ortheziidae) showing different new adaptations, such as laying eggs in a special cavity under the body or in a solid wax sac behind the body. In turn, some divergent Monophlebinae and their descendants (Phenacoleachiidae, Carayonemidae, and “primitive” neococcids) demonstrate again incomplete or complete ovoviviparity putting partly developed embryos inside the marsupium or laying fully developed embryos outside the body. Among neococcids, complete ovoviviparity of “primitive” mealybugs like *Puto*, *Rastrococcus*, *Paraputo*, *Heliococcus*, etc. (see above) evolves into incomplete oviparity (or almost normal oviparity) of some divergent mealybugs (like *Pseudococcus* Westwood, 1840, *Atrococcus* Goux, 1941 and others). “Primitive” soft scales (like Pulvinariini and Eriopeltinae) form a loose ovisac as in their faraway ancestors from Monophlebinae and Xylococcinae, but in contrast to the last, they use for the ovisac construction not multilocular pores, but tubular ducts of different structure. In turn, many divergent Coccidae and Kermesidae again lay partly developed eggs in a cavity under the body, that sometimes (in *Kermes* Boitard, 1828) looks like the marsupium of giant scales. The most aberrant and divergent families Asterolecaniidae s.l., Diaspididae and Phoenicococcidae s.l. again restore obligate complete ovoviviparity in many genera (Gavrilov-Zimin 2018).

**Figure 7.**
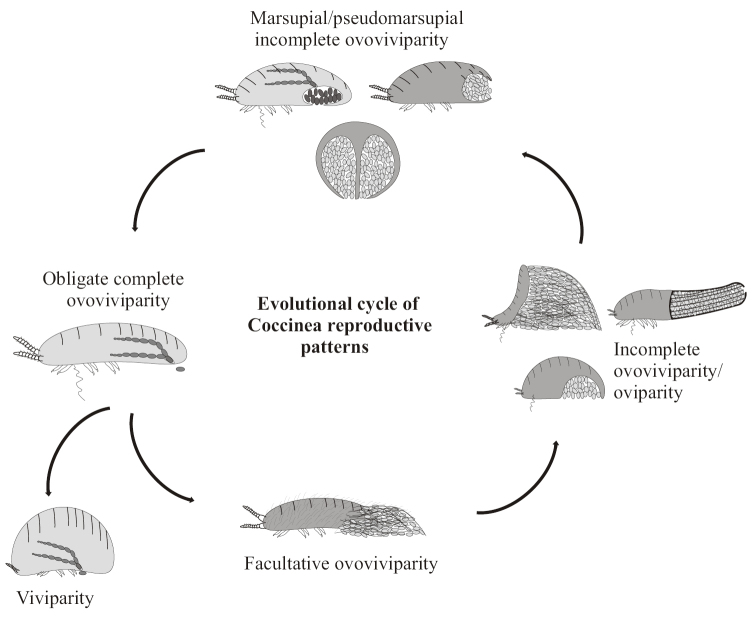
Cyclic evolutional conversions of reproduction pattern in Coccinea from oviparous to (ovo)viviparous variants with the emergence of new modes of eggs protection.

In the large order Heteroptera, examples of viviparity or ovoviviparity have been reported for the families Polyctenidae, Cimicidae, Anthocoridae, Plokiophilidae, Microphysidae, and also for some species of Aradidae and Lygaeidae (e.g. Hagan 1931, 1951; Carayon 1956, 1961). The most studied viviparous species of Heteroptera is the polyctenid *Hesperoctenesfumarius* (Westwood, 1874), an ectoparasite of bats. The oviduct of this bug does not have a spermatheca or any similar organ; during copulation the sperm pass directly into the lower part of the common oviduct, then migrate to the paired oviducts and pass through the walls of oviducts in the haemocoel. The ovulation, fertilization and at least part of the embryonal development of the egg occur in immature insects following, thus, a paedogenetic mode (Hagan 1931: 38, 1951: 396). The egg lacks chorion and yolk receiving the nutrition for embryonal development from the follicular epithelium of the mother’s body. At the stage of katatrepsis, the embryo forms peculiar structures, pleuropodial extensions, which grow and surround the embryo by a pleuropodial sheath (Fig. 8). This sheath, probably, plays a role of the placenta in the nutrition of the embryo (Hagan 1951: 400). A similar embryonal organ was also found in the viviparous *Physopleurellapessoni* Carayon, 1956 (Anthocoridae) (Carayon 1956: 109). In many other Anthocoridae and also in Cimicidae, eggs have a chorion and only a part of the embryogenesis occurs inside the mother’s ovary (Carayon 1961, 1966: 179) and so, incomplete ovoviviparity takes place. On the other hand, the absolute majority of Heteroptera demonstrate usual oviposition of the eggs prior to embryogenesis.

**Figure 8.**
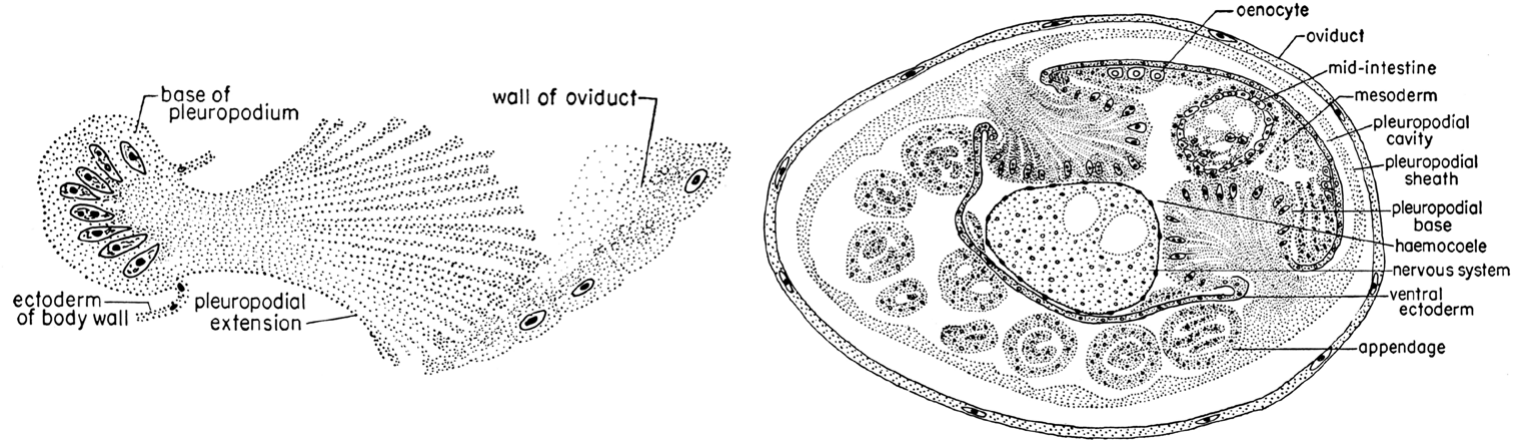
Pleuropodial extension (left figure) and pleuropodial sheath (right figure) in the embryo of viviparous *Hesperoctenesfumarius* (Heteroptera) (after Hagan 1931, 1951).

The very irregular distribution of (ovo)viviparous taxa among Paraneoptera (Fig. 9) (and among animals as a whole) denotes multiple and separate origins of this mode of reproduction in different phylogenetic lines. This is confirmed by all comparative studies of the problem (see, for example, the last reviews of Batygina et al. 2006; Ostrovsky et al. 2016). On the other hand, until recent times there was no clear understanding of the reasons for the emergence of ovovivipity/viviparity within originally oviparous taxa. During centuries, ecological reasons of viviparity origin have been considered as most likely (see for review: Hagan 1951). Some authors tried to associate the emergence of viviparity with the life in dry climate, others – with the life in wet climate, cold or hot environment, quality of food, passive or active mode of animal life, etc. Another common approach to the problem is the hypothesis of significant evolutionary advantages of viviparity, since developing embryos are protected by the mother’s body (see, for example, Hagan 1951; Meier et al. 1999; Ostrovsky et al. 2016 and large lists of references in these reviews). On the other hand, it seems that, in spite of the “adaptive advantage” hypothesis, both viviparous and ovoviviparous taxa are very few in nature in comparison with oviparous ones. Even among vertebrate animals, this mode of reproduction characterizes only mammals, small number of reptiles and fishes, whereas most vertebrates are oviparous. As for invertebrates, the (ovo)viviparous species comprise at best only several percent of the total number of species, being known as occasional occurrences in many large phyla. Moreover, viviparous taxa (these are usually occasional genera, rarely the whole families and very rarely higher rank taxa) are characterized by depressed taxonomic and morpho-anatomical diversity. Paraneoptera insects illustrate this situation especially clearly. This huge group of insects comprises about 115,000 species in the world fauna with only 5–6% of them being ovoviviparous or viviparous (Fig. 3), and most (> 5000) of these ovoviviparous/viviparous species of Paraneoptera are known in Aphidinea, i.e. in the group, which is characterized by very low morpho-anatomical diversity and includes species, genera and even families identified by metrical characters only. In the largest animal group, Coleoptera, comprising about 400,000 species, only occasional species from several families were found to be ovoviviparous or viviparous (Iwan 2000; B. Zilberman, personal communication). In the large insect order Diptera, comprising about 125,000 species, 61 events of independent origin of different variants of facultative/obligate ovoviviparity and viviparity were reported, including occasional species from different families as well as several small families with all species being viviparous (Meier et al. 1999). Gavrilov-Zimin (2018) hypothesized that evolutionary transformation of oviparity to ovoviviparity and, further, to true viviparity was an alternative way of phylogenesis, that occurs when usual oviposition comes into conflict with different morphological or physiological apomorphies of the ancestral species and its descendants. According to this hypothesis, most common reasons for the obligate egg retention are different variants of paedogenesis and neoteny, when the reproducing larva or nymph has lost special adult structures responsible for oviposition, with quick passage of the egg through the oviducts and fertilization of the egg in ectodermal parts of the oviduct (where a spermatheca is located). This presumption is rather clearly illustrated by Paraneoptera insects. Phylogenetic lineage Aphidococca is fully paedogenetic/neotenic; many viviparous true bugs and psocids have clear features of larvalization; all viviparous trips are known in the suborder Tubulifera, which is characterized by the loss of ovipositor, etc. A similar situation also occurs in the cases of different morphological or physiological transformations, which are related not with paedogenesis, but with changes in the imaginal reproductive system. Thus, as shown earlier by some authors (for example, Carayon 1961), parthenogenesis and change of fertilization location, from ectodermal to mesodermal parts of genitalia (up to the point of fertilization in vitellarium), are important preconditions to the origin of ovoviviparity/viviparity. The egg can start developing only after fertilization or when fertilization is not needed. If the egg is fertilized inside the vitellarium, it has enough time for the embryo to complete development before oviposition. For example, in viviparous/ovoviviparous true bugs (Heteroptera, Cimicoidea) of the families Anthocoridae, Cimicidae, Polyctenidae, and Plokiophilidae, the unique traumatic insemination and fertilization takes place; the male punctures the female body with its copulatory organ and injects sperm outside the female reproductive system. The insemination in this case is correlated with the structures of the so-called “paragenital system” consisting of “spermalege” (“organ of Ribago” or “organ of Berlese”), “seminal conceptacles”, “spermodes” and “syncitial bodies”, which is used for transporting and temporarily preserving the spermatozoa before their arriving into ovarioles (Carayon 1966).

**Figure 9.**
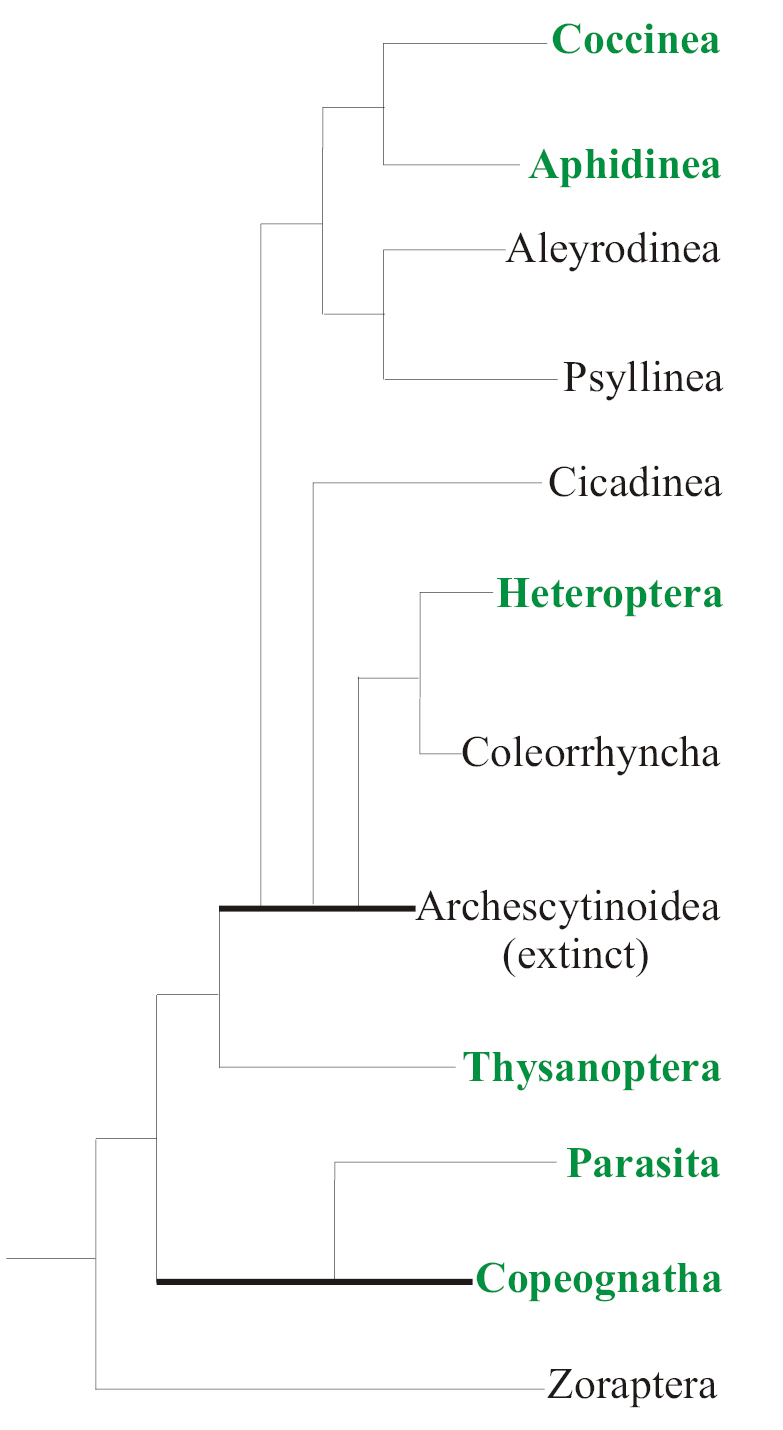
The phylogenetic tree of Paraneoptera based on Shcherbakov and Popov (2002), Kluge (2020), Gavrilov-Zimin (2020) with modifications. Higher rank taxa, including viviparous and ovoviviparous genera/families, are highlighted in green. Bold lines are used for paraphyletic taxa.
